# Supplementary material for: Gene Expression Within a Human Choroidal Neovascular Membrane Using Spatial Transcriptomics
Source: Invest Ophthalmol Vis Sci. 2023 Oct 25;64(13):40. doi: 10.1167/iovs.64.13.40 (PMC10615143; doi:10.1167/iovs.64.13.40)
Supplement: Supplement 1 [file iovs-64-13-40_s001.pdf]

GENE EXPRESSION WITHIN A HUMAN CHOROIDAL NEOVASCULAR MEMBRANE USING SPATIAL TRANSCRIPTOMICS

Andrew P. Voigt[1,2], Nathaniel K. Mullin[1,2], Emma M. Navratil[1,2], Miles J. Flamme-Wiese[1,2], Li-Chun Lin[3], Todd E. Scheetz[1,2], Ian C. Han[1,2], Edwin M. Stone[1,2], Budd A. Tucker[1,2], Robert F. Mullins*[1,2]

1. Department of Ophthalmology and Visual Sciences, the University of Iowa Carver College of Medicine, Iowa City, IA, 52242

2. Institute for Vision Research, the University of Iowa, Iowa City, IA, 52242

3. University of Iowa Neuroscience Institute, the University of Iowa Carver College of Medicine, Iowa City, IA, 52242

Correspondence:

Robert F. Mullins
4130 MERF
375 Newton Rd
Iowa City, IA 52242 USA
Phone: (319) 335-8222
Email: robert-mullins@uiowa.edu

Supplementary Figures

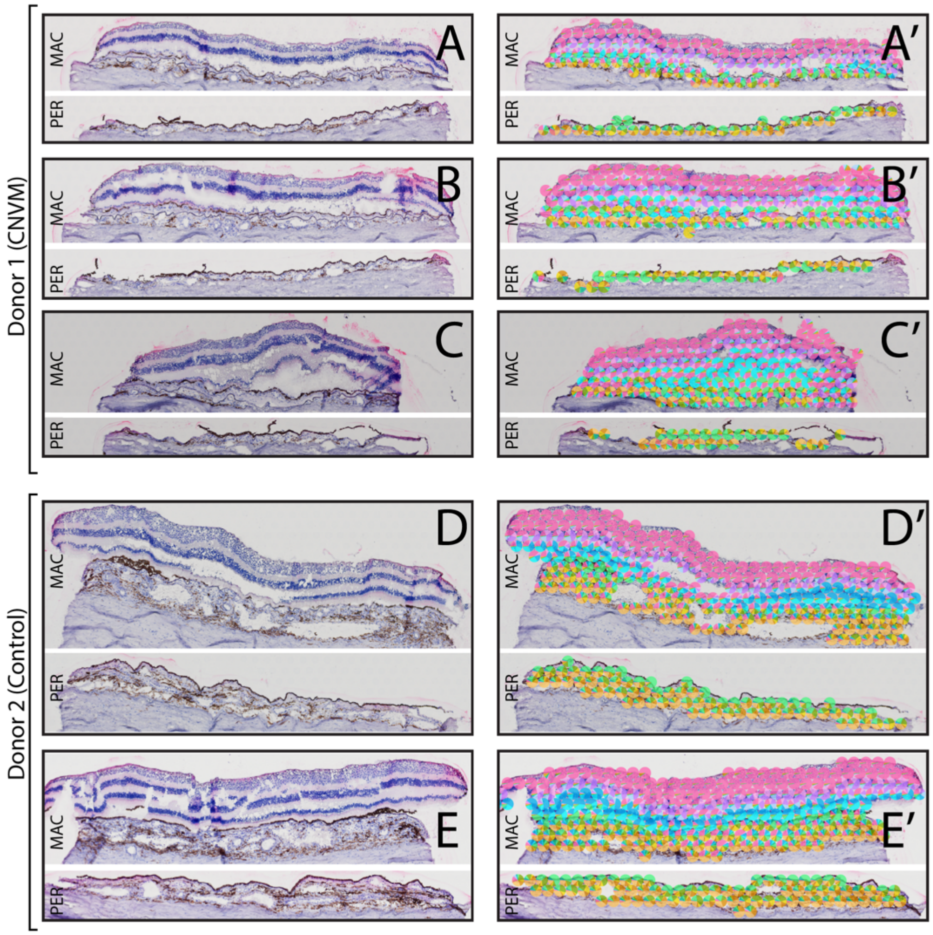

SI Figure 1: Three H&E tissue sections from the MNV donor (**A-C**) and two tissue sections from the control donor (**D-E**) are visualized. **A' – E':** The RNA contribution from retinal, RPE, and choroidal cell types was estimated in each spatially barcoded spot and displayed as a pie-chart as designated in Figure 2.

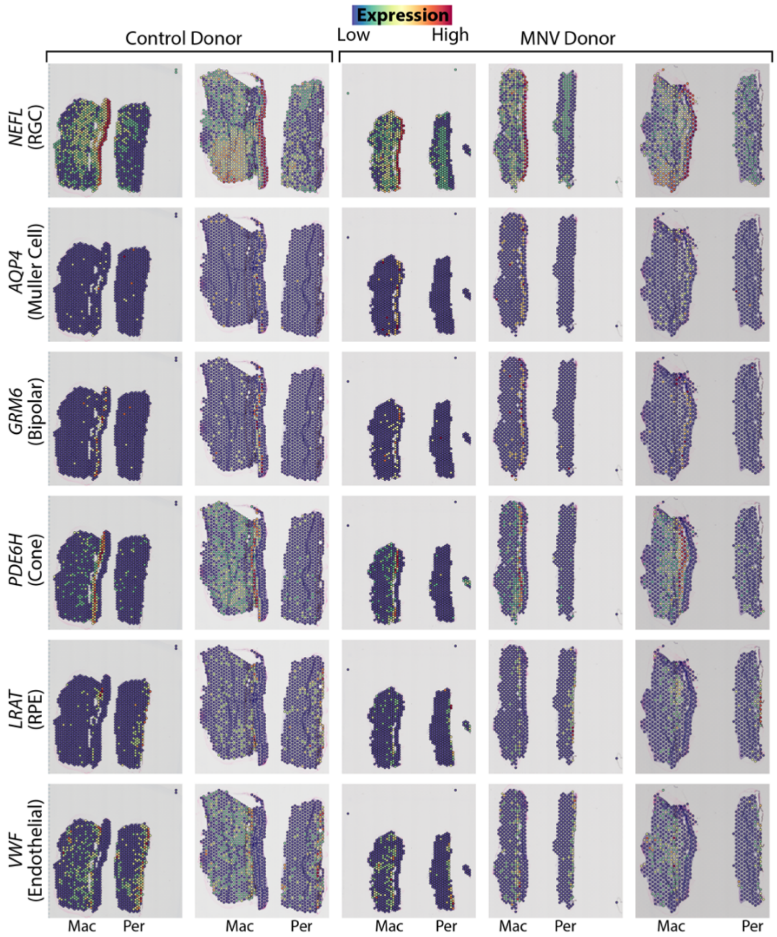

SI Figure 2: Spatial expression of hallmark retinal, RPE, and choroidal genes. Each row depicts the spatial expression of one gene, with a parenthetical comment of the cell population that most highly expresses that gene (according to previous single-cell RNA sequencing studies). Each column depicts one of the five sections used in the spatial transcriptomics experiments (columns 1-2 correspond to the control donor while columns 3-5 correspond to the MNV donor). Each 55-micron capture spot is outlined in black, and the color of the spot represents the detected expression (red corresponds to high expression while blue corresponds to low expression).

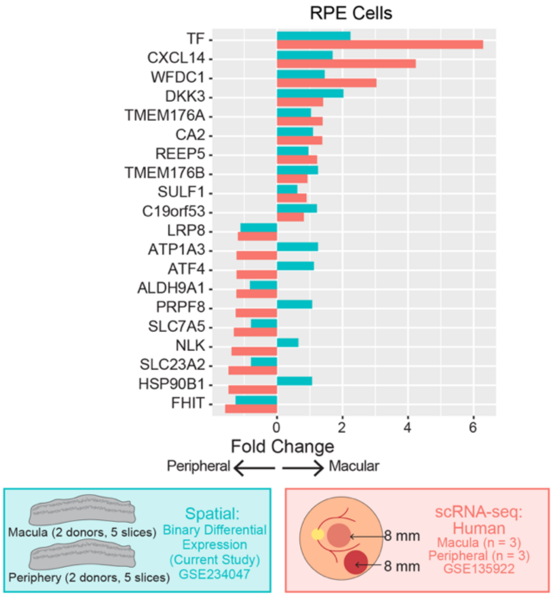

SI Figure 3: Comparison of macular and peripheral gene expression between spatial and single-cell RNA sequencing studies. Differential expression was completed to compare macular and peripheral enriched genes in the RPE. Cell type deconvolution and region were used as covariates for differential expression analysis. For RPE cells, the top differentially expressed genes between the macula and periphery were identified from a previous single-cell RNA sequencing study. Differential expression results were compared between this previous study (red) and the current spatial study (blue). Positive $\log_2$ fold-changes are associated with increased expression in the macula.
